# A Microbiological and Sensory Evaluation of Modified Atmosphere-Packed (MAP) Chicken at Use-By Date and Beyond

**DOI:** 10.3390/foods13132140

**Published:** 2024-07-05

**Authors:** Karin Söderqvist, Max Peterson, Marcus Johansson, Viktoria Olsson, Sofia Boqvist

**Affiliations:** 1 Department of Animal Biosciences, Swedish University of Agricultural Sciences, P.O. Box 7023, 750 07 Uppsala, Sweden; mxpe0001@stud.slu.se (M.P.); sofia.boqvist@slu.se (S.B.); 2 Department of Food and Meal Science, Kristianstad University, 291 88 Kristianstad, Sweden; marcus.jc.johansson@hkr.se (M.J.); viktoria.olsson@hkr.se (V.O.)

**Keywords:** shelf life, poultry, broiler, spoilage, discrimination triangle test, preference test

## Abstract

Consumers are responsible for a large proportion of food waste, and food that has reached its use-by or best-before date is often discarded, even if edible. In this study on fresh chicken, the suitability of use-by dates currently used in the EU was evaluated by using microbial and sensory analyses. This was carried out by analyzing bacterial populations of chicken breast fillets (*M. pectoralis major*) at three different time points (use-by date, 2 days past use-by date, 4 days past use-by date) and two different storage temperatures (4 °C, 8 °C). A discrimination triangle test was performed to check for sensory differences between chicken breast fillets cooked at the three selected time points for both storage temperatures. A consumer preference test was also performed for chicken breast fillets that had been stored at the highest recommended temperature (4 °C) and after being cooked at the three time points. Changes in populations of total aerobic count (TAC), *Enterobacteriaceae* (EB), and lactic acid bacteria (LAB) were recorded over time. Despite large differences in bacterial counts at the selected time points, with TAC populations of approximately 6.5 and 8.0 log CFU/g at use-by date and four days after use-by date, respectively, storage for two or four extra days had no significant effect on the sensory parameters of cooked chicken compared with chicken consumed at its use-by date. Since the TAC populations were close to or above levels that are associated with spoilage, more work is needed to explore if shelf life can be extended.

## 1. Introduction

Enormous volumes of food waste are generated worldwide, and reducing this waste stream provides an obvious opportunity to contribute to food security and a more sustainable food system [1,2]. The consumption stage is responsible for the largest proportion of food waste generated for most food groups within the European Union (EU), and it has been reported that more than 50% of all food discarded at consumption level and 80% of food discarded at retail level is still edible at the time of discarding [3,4]. Food waste in the latter stages of the production chain (closer to consumption point) contributes to the current food system being highly unsustainable, mainly due to waste of economic and natural resources.

Under EU regulations, fresh chicken meat must be labeled with a use-by date, and food that has passed this date must be discarded (Regulation No. 543/2008 and 1169/2011). This is not the case for other types of fresh meat, such as pork and beef. This discrepancy is likely due to differences in slaughter techniques, with greater challenges controlling bacterial contamination during slaughter of broiler chickens [5]. Poultry meat is known to have a higher initial bacterial contamination level than, e.g., beef or pork, and is thus a fast-perishing product [6]. Fresh poultry products are usually packaged in modified atmosphere packaging (MAP), providing an anaerobic environment consisting of approximately 30% CO_2_ and 70% N_2_, as a way of prolonging shelf life [7,8]. Even with MAP and cool storage at <4 °C, the shelf life of chicken meat is short, usually with only 10 days from slaughter to use-by date. The rate of microbial spoilage is, however, dependent on the contaminants involved and in particular the specific spoilage organisms (SSOs) that originate from the raw materials or the processing environment. These organisms only represent a small fraction of the initial microbiota, and their growth is affected by the storage conditions for each product package [9]. Labeling with use-by date likely contributes to unnecessary waste of fresh chicken products, e.g., products with relatively low numbers of SSOs that are stored at low temperatures. To reduce food waste, there is therefore a need for a system change, in which the date label aligns with the actual quality of the food product. This should be performed without compromising the safety of the food, since pathogenic bacteria associated with the consumption of fresh chicken meat, such as *Campylobacter jejuni*, do not grow at refrigerator temperatures and will thus not increase with an extended shelf life [10]. 

Screening for total aerobic counts (TACs) and *Enterobacteriaceae* (EB) is often used by Food Business Operators to evaluate process hygiene and quality or expected shelf life of their products [5]. These analytical parameters are, however, highly unspecific and will not depict the complex food microbiota that will ultimately cause spoilage. Nevertheless, poultry meat is considered to be spoiled when TAC levels exceed 10^7^ CFU/g [11]. Typical spoilage bacteria associated with MAP fresh chicken products include lactic acid bacteria (LAB), *Brochothrix thermosphacta*, and genera such as Serratia, Shewanella, and Hafnia [5,12]. 

The aim of this study was to evaluate the suitability of use-by dates currently used in the EU for chicken breast fillets (*M. pectoralis major*) in MAP, or specifically if the shelf life of this product can be extended. The evaluation was based on sensory aspects, populations of TAC, EB, and LAB, and identification of the most prevalent bacterial species in chicken breast fillets at use-by date (approximately 10 days after slaughter) and at 2 and 4 days after this date when stored at 4 °C (recommended maximum storage temperature) or 8 °C (slight abuse temperature) throughout their shelf life.

## 2. Materials and Methods

The evaluation comprised two substudies: (i) microbiological analysis of the raw chicken fillets and (ii) sensory evaluation of cooked chicken in a preference test and a discrimination triangle test.

### 2.1. Study Design and Sample Preparation

Chicken breast fillets packaged in MAP (30% CO_2_, 70% N_2_) produced by one of Sweden’s market leaders in chicken products were obtained from a local supermarket in Uppsala, Sweden. Each package contained two fillets, and each fillet weighed around 150–200 g. For each of five trials in total, 18 packages originating from the same batch, i.e., chicken from the same broiler slaughterhouse and with the same use-by date, were retrieved (*n* = 90 in total). These were transported chilled to the Food Safety Laboratory at the Department of Animal Biosciences, Swedish University of Agricultural Sciences (SLU, Uppsala), on the day on which they were delivered to the supermarket from the slaughterhouse (6–8 days before use-by date; total shelf life 10 days). Half of the packages from each batch (*n* = 9) were placed in an incubator set at 4 °C and the other half in an incubator set at 8 °C, and kept there until analysis. In total, 86 packages of chicken breast fillets were subjected to analysis (four packages were excluded due to mismatching use-by dates or visible signs of spoilage).

**Figure 1:**
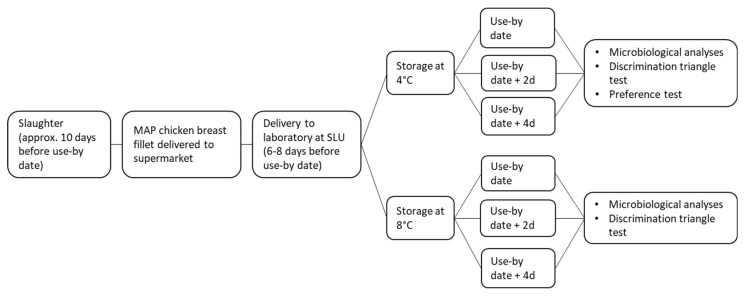
Flowchart showing the different analyses performed in the study.

Batches of six packages, three from 4 °C storage and three from 8 °C storage, were analyzed on the use-by date (day 0) and at 2 and 4 days of storage after the use-by date (see Figure 1). One fillet from each package was used in microbiological analysis, and the other fillet was vacuum-packed and kept frozen at −20 °C for use in sensory evaluation tests within 2 months. According to Augustyska-Prejsnar [13], short-term freezing at −20 °C does not markedly affect the sensory properties of chicken meat.

The microbiological analysis was designed to detect a difference of 0.5 log CFU/g to a power of 80%. Assuming a standard deviation of 0.4 [7], in each trial (replicate packages from the same temperature and storage time), a sample size of 11 would be required to achieve this aim. A sample size of 15 was selected, to allow triplicate analyses in the five trials.

### 2.2. Microbiological Analysis and Identification of Isolates

From each chicken fillet, a 25 g sample of surface meat was collected aseptically with scissors and tweezers and transferred to a stomacher bag with 225 mL of 0.1% Peptone water solution tempered to 25 °C. The bag was then placed in a stomacher, and the sample was homogenized for 120 s at room temperature (AES Laboratoire Easymix, bioMérieux, Craponne, France). A dilution series was performed using Dilucups (Dilucups^®^ Elegance; LabRobot, Stenungsund, Sweden) and appropriate dilutions of each sample were applied to Petrifilms (3M Petrifilm™, St. Paul, MN, USA). Petrifilms for EB count were incubated at 37 °C for 24 ± 2 h, while Petrifilms for TAC and LAB were incubated at 30 °C for 48 ± 4 h. After incubation, colonies on all Petrifilms were enumerated according to the interpretation guide for each parameter. Bacterial counts were expressed as logarithmic values.

During the fourth and fifth trials in the microbiological substudy, bacterial identification was performed on 24 samples representing both storage temperatures (4 and 8 °C) and the three selected time points (days 0, 2, and 4 after use-by date). Samples were collected 2 and 4 days after the use-by date from the fourth trial and from days 0 and 2 from the fifth trial, thus with day 2 represented twice. The Petrifilms for TAC representing the highest dilution were selected to represent a subsample of the most common colonies on each of these samples. Colony material from five colonies was collected from each of these Petrifilms and spread separately onto bovine blood agar plates, which were incubated at 30 °C for 48 h and then stored at 4 °C for up to five days. For analysis, colonies from the blood agar plates were collected with toothpicks and applied in duplicate onto metal 96-well plates customized for matrix-assisted laser desorption/ionization-time-of-flight mass spectrometry (MALDI-TOF-MS) (Bruker Daltonics GmbH, Karlsruhe, Germany). A 1 µL portion of HCCA matrix (α-cyano-4-hydroxycinnamic acid dissolved in Bruker^©^ standard solvent containing acetonitrile, water, and trifluoroacetic acid) was added to each well. The wells were left to dry out before placing the plates in the MALDI apparatus. Since a Biotyper score of ≥2.0 is considered accurate and confirmatory for bacterial genus and species according to the manufacturer’s standard interpretation criteria, only bacterial organisms with a score of 2.0 or above were recorded.

### 2.3. Sensory Evaluation Sample Preparation

Prior to the sensory evaluation, a pilot study of cooking method and time was performed. Sous-vide cooking was deemed the best cooking method since it facilitates the best control over the inner temperature of the cooked chicken fillets. The target temperature was set to the recommended inner temperature of boneless chicken meat, 72 °C [14]. Results from the pilot study showed that sous-vide cooking at 72 °C for 1 h was adequate to reach the desired core temperature while not overexposing the samples to the heat treatment. The chicken breast fillets were thawed overnight in a fridge set to 8 °C and then cooked in their individual vacuum bags. Fillets were then sliced into pieces of 10 g each and placed in petri dishes labeled with three-digit codes.

#### 2.3.1. Preference Test

**Figure 2:**
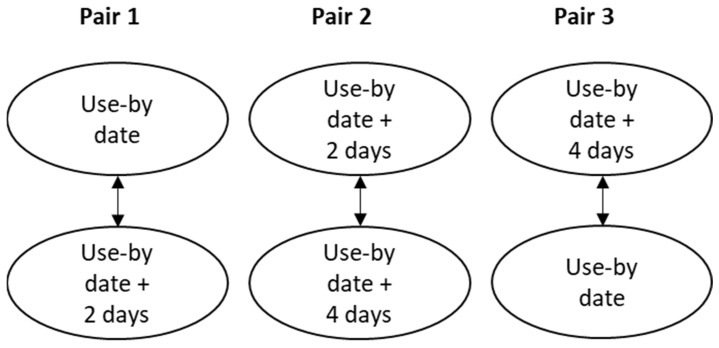
Illustration of pairwise comparisons included in the preference test.

A paired preference test was performed which involved 45 test participants. These were mainly staff and students who volunteered on one occasion in the canteen at the Faculty of Veterinary Medicine and Animal Science, Swedish University of Agricultural Sciences. The number of individuals included was based on the number of fillets available from the batches investigated microbiologically. Chicken breast fillets from time points 0, 2, and 4 days after use-by date, stored at 4 °C (see Figure 1), were used in this test. All time points were tested against each other, resulting in six samples for each participant to assess in three pairwise comparisons, as shown in Figure 2. The data were collected using EyeQuestion software (version 4.11.68, Logic8 BV, Elst, The Netherlands) which was also used to randomize the order of the pairs, and of the samples within pairs, for each test participant. The test participants were asked to evaluate two samples at a time and record which sample they preferred based on overall liking of the chicken meat; the participants were also able to comment on their choice in free text.

#### 2.3.2. Discrimination Triangle Test

A discrimination triangle test was performed at the Department of Food and Meal Science, Kristianstad University, in a sensory laboratory designed according to ISO 8589 [15]. In this test, eight selected panelists trained according to ISO 3972 [16], ISO 8586-1 and ISO 8586-2 [17] guidelines evaluated whether they could perceive a difference in taste between chicken fillets prepared as described above. The number of panelists included was based on the available amount of fillets and study logistics. All time points, 0, 2, and 4 days after use-by date, and both storage temperatures, 4 °C and 8 °C, were used in this test. This resulted in 15 triangles that were assessed in duplicate by each panelist. The panelists received three samples at a time, two identical and one that differed, each with individual three-digit codes. Sample order within the triangles along with serving order of the triangles was randomized for each panelist using EyeQuestion software. The panelists were asked to choose one sample per triangle that they believed differed in flavor, odor, and/or texture. They were also able to leave comments on their choice. Between assessments, panelists were instructed to rinse their pallet using water and neutral crackers. Assessments were performed during two consecutive 30 min sessions with a 15 min break in between.

### 2.4. Statistical Analysis

Results from the microbiological analysis were re-calculated as mean value of the triplicates within each sampling time, and the differences between means were analyzed by ANOVA in RStudio^®^ (Version 4.2.0, RStudio Team (2020), Boston, MA, USA). QQ-plots for each bacterial type (TAC, EB, and LAB) were used to compare shapes of distribution of pairwise datasets using RStudio^®^. The plots indicated that the data were normally distributed. Tukey’s HSD-test in RStudio^®^ was used to identify whether the groups were significantly different from one another.

Pairwise comparisons for each participant in the preference test were performed using EyeOpenR^®^ (version 4.11.68, Logic8 BV, Amsterdam, The Netherlands). A binomial distribution test was used to analyze results from both the preference test and the discrimination triangle test in EyeOpenR^®^. A significance level of 95% was used for all tests.

## 3. Results and Discussion

**Figure 3:**
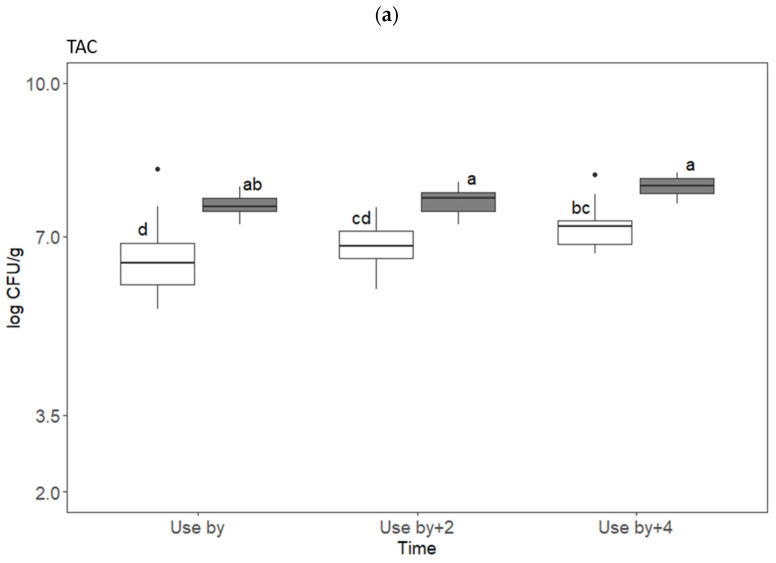

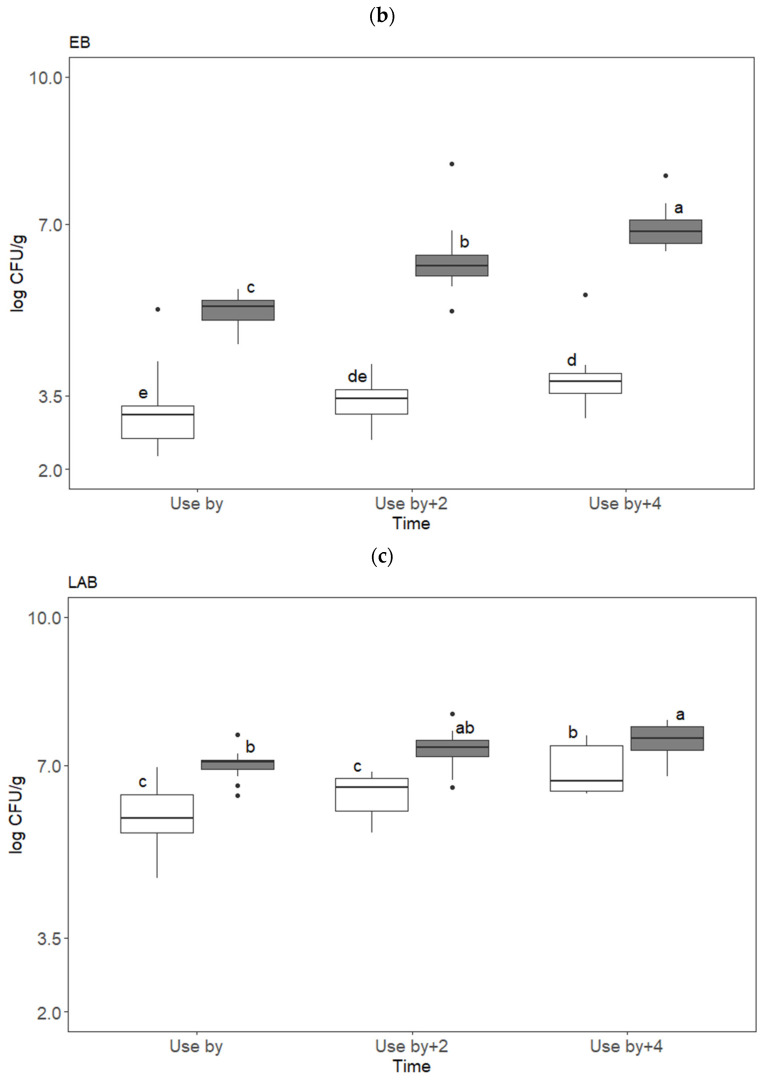
Populations (log CFU/g) of (**a**) total aerobic counts (TACs), (**b**) *Enterobacteriaceae* (EB), and (**c**) lactic acid bacteria (LAB) in chicken breast fillets samples from five batches stored at 4 °C (white boxes) and 8 °C (grey boxes). Different letters indicate statistically significant differences between groups in each diagram. Dots represent outliers in the microbiological analyses.

Contrary to expectations, the trained panelists in the discrimination triangle test found no significant difference between, e.g., chicken fillets stored at the higher temperature for the longest time and fillets stored at the lower temperature for the shortest time. This result was supported by the outcome of the preference test in which it was found that there was no significant difference in liking of chicken fillets that had been stored at 4 °C until use-by date or 2 or 4 days after that date. Even though there were no signs of sensory changes, the bacterial populations of TAC, EB, and LAB in fresh chicken breast fillets increased with storage duration (Figure 3).

As expected, there were significantly higher populations of TAC, EB, and LAB in fillets that had been stored at 8 °C during their shelf life than in fillets stored at 4 °C. There was a large increase (approx. 2–3 log) in EB populations when chicken fillets were stored at 8 °C rather than 4 °C (Figure 3). However, the number of LAB at the different time points in chicken fillets stored at 8 °C only increased by 0.6–1.0 log CFU/g compared with fillets stored at 4 °C, with total LAB counts ranging from 6.0 to 7.5 log CFU/g at both temperatures. It has been suggested that meat spoilage occurs when TAC populations reach around 7 log CFU/g [5,18,19]. In the present study, the populations of TAC were close to 7 log CFU/g already at use-by date when stored at 4 °C, which is the recommended maximum storage temperature for chicken breast fillet, with some samples exceeding this level (Figure 3). The highest log CFU/g value of TAC detected for samples that had been stored for four extra days at 4 °C was 7.2 (Figure 3). Thus, the TAC levels were remarkably high, even for chicken breast fillet that had been stored according to the recommended temperature and length of shelf life as indicated on the pack label. Despite this, based on the results of the sensory evaluation including the comments given by the panelists, there was little to indicate that chicken at use-by date and after extended shelf life was of unacceptable quality. Some of the participants explained their choices in the preference test, stating that the sample they preferred was, e.g., juicier, less dry, tasted better, or was more tender. The most common comment (*n* = 15) was “Did not perceive any difference” (or similar), and this comment was found for all three pairwise comparisons. There were only two negative comments about how the chicken meat tasted: “Don’t like either” and “Both were a little bit disgusting”.

For samples that were stored at 8 °C, which is a higher temperature than recommended for fresh meat, the TAC population reached levels between 7 and 8 log CFU/g in all samples already at use-by date. While storage at 8 °C is neither ideal nor recommended, it was included in the study design since temperatures above 4 °C are often found in home refrigerators [20,21,22]. The highest log CFU/g value of TAC detected for samples that had been stored for four extra days at 8 °C was 8.0 (Figure 3), which is a level that has been reported to cause noticeable odor changes [23]. Metabolites which cause the off-flavors and off-odor are produced by SSOs, which compose a varied fraction of the TAC microbiota. Therefore, TAC populations can be high in food without signs of spoilage and should not be used as the only indicator of spoilage [24]. The discrimination triangle test performed was designed to evaluate whether samples were perceptibly different but did not identify the attribute(s) which differed. Comments on the choices can sometimes give an indication, and in this test there were no comments indicating that the chicken meat evaluated was of unacceptable quality. The panelists who commented on their choice wrote descriptions such as “a little bit sweeter”, “milder taste”, “an intense note of grass”, “less acidity”, “dryer”, “softer consistency”, and “juicier” when referring to the sample that they thought differed from the other two. However, comments were only given for 17 out of a total of 240 discrimination triangle tests performed, and the comments referred to the sample that actually differed from the other two in only six of these cases. Only in one comparison was a significant difference (*p* = 0.016) between the chicken fillets recorded by the panelists for samples that had been stored at the same temperature (4 °C), but with a different shelf life (one had been stored for 2 extra days after use-by date and the other for 4 extra days). There is no logical explanation why these fillets were perceived as different, since they had similar populations of TAC, EB, and LAB. In the same comparison made by participants in the preference test, there was no significant difference in liking, although different individual fillets were used in the two different sensory evaluations. Discrimination triangle tests are designed to expose sublime differences that may not be apparent in consumer evaluations, but in trying to explain the different results, one theory may be that some chicken fillets included in the discrimination triangle test had a higher abundance of certain SSOs that were not analyzed in this study but which altered the sensory characteristics. 

Identification of the most prevalent bacterial species in chicken breast fillets was performed as a screening investigation but also to enable evaluation of potential correlations between certain bacteria and findings in the sensory studies. In total, 120 pure-cultured isolates from two batches of chicken samples were analyzed with MALDI-TOF-MS, representing samples from both storage temperatures at use-by date and 2 and 4 days after use-by date. The colonies analyzed were recovered from highly diluted sample material, which means that the species identified were likely to be among the most prevalent culturable bacteria in the samples. Seven bacterial species were identified by MALDI-TOF-MS. *Carnobacterium* was found in at least one out of five tested colonies from all samples, and the species identified was either *C. divergens* (*n* = 19) or *C. maltaromaticum* (*n* = 17). *Brochothrix thermosphacta* was identified in 10 samples. *Carnobacterium* spp. and *B. thermosphacta* are associated with food spoilage and are frequently predominant elements of the microflora in chilled MAP meat products [19,25,26]. Other species identified were *Hafnia alvei* (*n* = 6), *Serratia proteamaculans* (*n* = 5), *Shewanella baltica* (*n* = 2), and *Yersinia ruckeri* (*n* = 1). There was no clear pattern in occurrence of certain bacterial species linked to a particular time or temperature except for *S. proteamaculans* and *H. alvei*, which were only isolated from samples of breast fillets stored at 8 °C. These bacterial species are reported to be commonly occurring in meat and chicken products during spoilage [19,27].

Within the EU, the use-by/best-before date for different food products is determined by Food Business Operators, based on experiences acquired through laboratory testing or recommendations provided in industry-developed guidelines [24]. Several factors at the production, retail, and household levels could affect bacterial spoilage and sensory characteristics, and thereby the shelf life of raw chicken meat. For example, some batches will have better initial microbiological quality, and storage at lower temperatures (than recommended) will suppress microbial growth, which means that shelf life will differ even between individual packages since they are likely stored at different temperatures. It has also been shown that storage temperature in home refrigerators and in retail refrigerators may be higher than the recommended level [20,21,22], so spoilage can occur even before the use-by/best-before date. The fixed use-by date set by the producer as the “date of minimum durability” (in Sweden approx. 10 days after slaughter) does not take any of these factors into account. Most fresh food products are labeled with a best-before date, to inform the consumer about how long the food can be presumed to be of optimum quality. Foods can often be consumed after this date, and consumers are encouraged to do so through campaigns such as “Look, smell, taste, don’t waste”, in order to reduce food waste [28]. Innovative alternatives, such as dynamic food labeling, have also been described. These are reported to give a more flexible indication of when a food item becomes inedible, e.g., via a system based on detecting a certain level of volatile organic compounds (VOCs) produced by SSOs in the food package, thereby indicating when the meat is no longer of good quality [29,30]. In this study, there were no significant differences in sensory aspects of cooked chicken breast fillets consumed at the use-by date or up to four days after the use-by date, which suggests that the current shelf life could be extended or based on a more flexible labeling system. However, a current barrier to introduction of a best-before date or a more flexible food labeling system (e.g., dynamic labeling) for fresh chicken meat is that it must be marked with a “use-by” date or “date of minimum durability” under EU regulation (EC) 543/2008 and cannot be sold after this date (EU 2011/1169, 543/2008). Consumers are also advised not to eat foods that have passed their use-by date, since these may pose a risk to human health. Raw chicken is highly perishable and can be easily spoiled, which will eventually affect the quality of the food item. However, the safety of raw chicken is not automatically worsened after the use-by date since the populations of bacterial pathogens associated with raw chicken, e.g., *Campylobacter* and *Salmonella*, do not increase during shelf life at recommended temperature [10,31]. Future studies could investigate if it would be possible to change to a best-before date or introduce dynamic food labeling for fresh chicken to reduce food waste, i.e., discarding of fully edible meat simply because the product has reached its use-by date. However, there is a trade-off between spoilage and food waste that needs to be considered. It should be noted that the present study only examined the sensory aspects of cooked chicken fillets, and not raw chicken, e.g., consumer reaction to odor when opening a package of chicken breast fillets some days after the use-by date. More research is needed on this important aspect of the product, and its correlation to bacterial population, since consumers will most likely not cook chicken fillets with off-odor.

## Data Availability

The original contributions presented in the study are included in the article, further inquiries can be directed to the corresponding author.
